# The Off-Targets of Clustered Regularly Interspaced Short Palindromic Repeats Gene Editing

**DOI:** 10.3389/fcell.2021.718466

**Published:** 2021-09-17

**Authors:** Manuel M. Vicente, Miguel Chaves-Ferreira, João M. P. Jorge, João T. Proença, Vasco M. Barreto

**Affiliations:** DNA Breaks Group, NOVA Medical School (NMS), Centro de Estudos de Doenças Crónicas (CEDOC), NOVA University of Lisbon, Lisbon, Portugal

**Keywords:** CRISPR/Cas9, off-targets, Cas9 variants, DNA repair, gene editing

## Abstract

The repurposing of the CRISPR/Cas bacterial defense system against bacteriophages as simple and flexible molecular tools has revolutionized the field of gene editing. These tools are now widely used in basic research and clinical trials involving human somatic cells. However, a global moratorium on all clinical uses of human germline editing has been proposed because the technology still lacks the required efficacy and safety. Here we focus on the approaches developed since 2013 to decrease the frequency of unwanted mutations (the off-targets) during CRISPR-based gene editing.

## Introduction

Since 2013, the field of gene editing is undergoing a revolution triggered by landmark publications showing that a ribonucleoprotein involved in the protection of bacteria from bacteriophages can be used both *in vitro* (Gasiunas et al., 2012; Jinek et al., 2012) and in human cell lines (Cong et al., 2013; Mali et al., 2013) to introduce double-strand breaks (DSBs) in DNA at precise locations. This remarkable defense mechanism is made of two main components, namely, a locus with a peculiar structure known as “clustered regularly interspaced short palindromic repeats” (CRISPR), discovered by Mojica et al. (2000) in 20 different microbes in 2000, and a set of CRISPR-associated (Cas) proteins. In a process known as adaptation, the Cas proteins capture the genetic information (the “spacers”) from invading bacteriophages and incorporate them into the CRISPR locus. In the CRISPR system that has been most frequently used for gene editing, the transcript of the CRISPR locus (pre-crRNA) is processed into CRISPR-RNAs (crRNAs) that will associate with a partially complementary trans-activator RNA (tracrRNA) and a Cas endonuclease, thus generating a ribonucleoprotein complex. The crRNA will hybridize to a complementary sequence in the target DNA named protospacer, provided this sequence is flanked by a 2–6 base pair protospacer adjacent motif (PAM) recognized by the nuclease, and the Cas endonuclease will then introduce a DSB at a precise location in the DNA. The targeting of specific sequences via spacers is termed interference.

In eubacteria and notably in archaea, the CRISPR systems are widespread. Three types of systems have been identified, each defined by a signature *Cas* gene: *Cas3* in type I systems, *Cas9* in type II, and *Cas10* in type III. There are excellent articles on the many ways bacteria have diversified their unique adaptive immune system (e.g., Makarova and Koonin, 2015); here, we discuss mainly the components of type II, which, due to its simplicity, has been the basis for most gene editing approaches. For editing purposes, the system was further simplified by the fusion of crRNA with the tracrRNA, generating a molecule known as single guide RNA (sgRNA) (Jinek et al., 2012). Thus, a sgRNA and the Cas9 in the cell are sufficient to introduce a DSB.

Multiple articles have alluded to the path from the early recognition of CRISPR loci as a set of adaptive immunity instructions, by Francisco Mojica, to the 2020 Nobel Prize in Chemistry given to Jennifer Doudna and Michele Charpentier for their repurposing of CRISPR components as a gene-editing tool (e.g., Lander, 2016). The explosion of publications using this flexible, simple, and relatively inexpensive tool and recent clinical applications based on CRISPR gene editing demonstrate the impact of this technological breakthrough, but problems remain. Depending on the context, the technique may lack the necessary efficiency and sequence specificity to mediate flawless editing. These concerns are particularly serious in the case of clinical applications using edited somatic cells (e.g., Lu et al., 2020; Frangoul et al., 2021) and notably in the case of heritable genome editing in humans, i.e., changes introduced in sperm, eggs, or embryos to make genetically modified children, which has already happened at least once despite the legislation in many countries barring all clinical uses of germline editing; early concerns due to the simplicity and potential of the technique followed by a call for a moratorium on germline genome editing signed by prominent figures in the field illustrate the ongoing fear (Baltimore et al., 2015; Lander et al., 2019).

**FIGURE 1:**
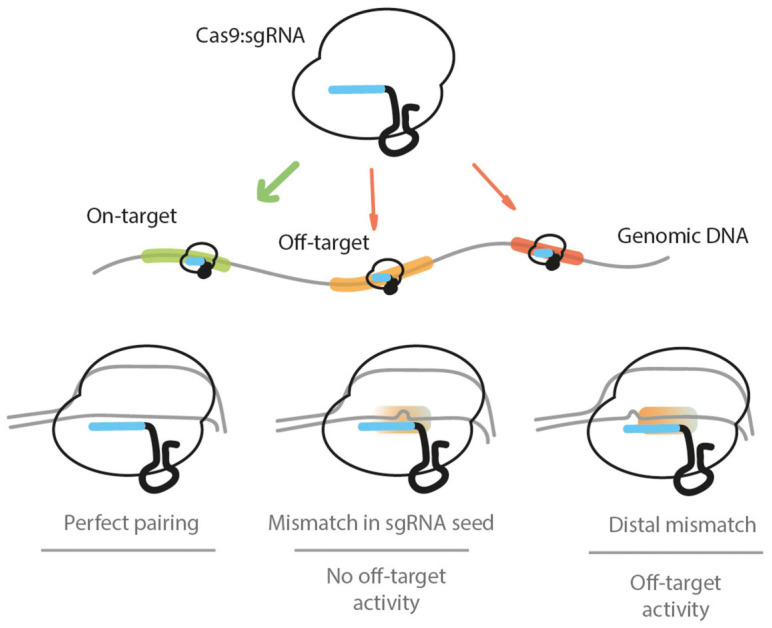
The Cas9:sgRNA ribonucleoprotein complex targets the genomic DNA when present in the cell nucleus. It will target PAM-adjacent sequences and may encounter several potential recognition sites. If there is a perfect base pairing between the target DNA and the sgRNA, a DSB is induced by Cas9. If there is a mismatch between the sgRNA and the DNA sequences present within the first 7–12 bp (proximal to the PAM) segment, the base pairing will not be sufficient to induce a DSB. If one or more mismatches between the DNA and sgRNA sequences are found in the PAM-distal (5′ end of the sgRNA) nucleotides, a DSB can be induced by Cas9, leading to off-target activity. The scenario of sgRNA sequence-independent off-targets is not depicted.

**FIGURE 2:**
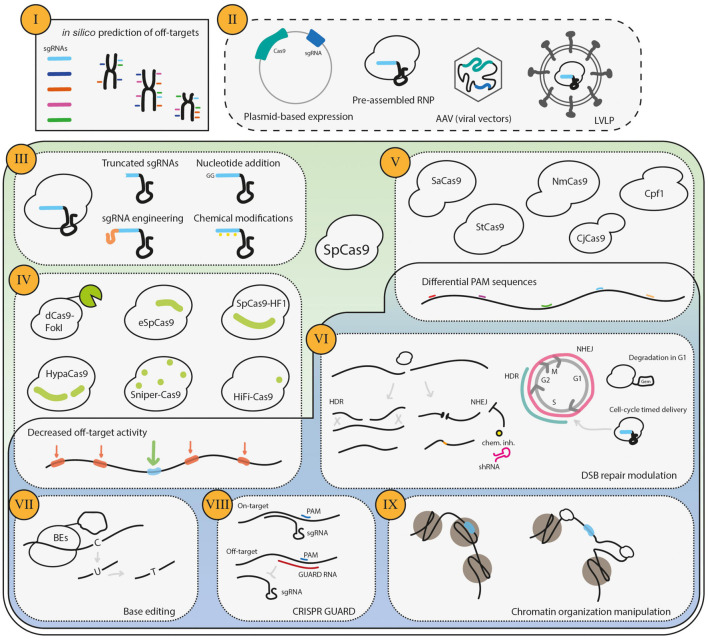
Strategies to decrease CRISPR/Cas9 off-target activity. The procedures to mitigate undesired CRISPR/Cas9 gene editing start **(I)** *in silico*, namely in the design of the sgRNAs. Several bioinformatic tools are available to determine the predicted off-targets of a given sgRNA, and one should choose a sequence that has high theoretical on-target activity and a low probability of generating off-target DSBs. The second step is **(II)** the delivery type of the CRISPR/Cas9 system players, which determines how long the cell becomes exposed to the RNP complex. Furthermore, **(III)** the engineering of the sgRNAs is an efficient strategy to decrease off-target activity. One additional major focus has been to **(IV)** engineer the highly specific SpCas9 forms with decreased off-target activity. Moreover, **(V)** the SpCas9 orthologs have a repertoire of PAM specificities, which increases the options for suitable sgRNA and may also decrease the off-target activity if the complexity of the PAM is higher than that of SpCas9. **(VI)** Cas9-mediated DSBs may be repaired by the HDR or NHEJ pathways, which generate distinct repair products. As the HDR repairs the DNA damage restoring the original sequence, it has been argued and shown that the repair of off-targets by this pathway does not generate undesired gene edits. HDR repair is promoted either by pharmacological inhibition or cell-cycle-regulation. More recently, the use of Base Editors **(VII)**, where a catalytically dead Cas9 or a Cas9 nickase is fused to a cytidine deaminase that drives a single nucleotide modification, has the potential to reduce off-targets, due to sequence requirements and the lack of DSBs. The blockage of off-targets by GUARD RNAs **(VIII)** has also been shown to reduce off-targets of Cas9. Finally, **(IX)** the chromatin organization manipulation may skew the off-target activity.

Here we will focus exclusively on one of the main problems of the technique: the unwanted changes in the genetic information that the CRISPR editing produces outside of the target region(s) (Figure 1). These undesired alterations are known in the field as “off-targets.” We will review why off-targets are observed when Cas9-mediated gene editing is used, the several strategies developed to decrease them (Figure 2), and the off-targets of other competing editing technologies, providing quantitative comparisons when the data are available.

## Clustered Regularly Interspaced Short Palindromic Repeats/Cas9 On- and Off-Target Activity

Most CRISPR-based editing involves DNA repair. Targeted DSBs were found to increase the integration frequency of reporters with homology arms by orders of magnitude (Rouet et al., 1994), which has been explored by several gene-editing techniques based on programmable nucleases, of which CRISPR editing is the latest example. In the case of knockins, the DSB will recruit the high-fidelity homology-directed repair (HDR) machinery to integrate a piece of genetic information introduced in the cell as a template (Heyer et al., 2010). In the case of a knockout, no template is introduced, and most strategies are based on the action of the error-prone non-homologous end-joining (NHEJ), which can repair the DSB in a flawed way, introducing deletions or insertions (indels) (Pardo et al., 2009) that lead to frameshifts and, consequently, transcripts that are targeted by the non-sense-mediated mRNA decay (NMD) surveillance pathway.

Unfortunately, the crRNA pairing with the protospacer only involves 20 base pairs and lacks the necessary complexity to always ensure a single hit in a genome with more than 3 billion base pairs, such as the human genome. Adding to the problem, the pairing of the 20-bp region does not require 100% identity and tolerates mismatches, which vastly increases the number of potential off-targets (Figure 1). Several studies have focused on the promiscuous nature of the binding of the Cas9:sgRNA complex to DNA, providing mechanistic insights on the off-target Cas9 activity. Early work on human cells revealed that a DSB could occur even when the PAM-distal region of the protospacer has up to five mismatches with the sgRNA (Fu et al., 2013; Hsu et al., 2013). Since these initial studies, it has been further found that the specificity of Cas9 is heavily determined by the first 7–12 bp-long seed sequence proximal to the PAM (Jiang and Doudna, 2017). Crucially, the DNA interrogation mechanism by Cas9 was later elucidated, revealing the PAM’s absolute requirement for double-stranded DNA unwinding, and the posterior evaluation of sequence similarity between the target sequence and sgRNA. These molecular insights made it clear that off-target activity should only be encountered in PAM-adjacent sequences (Sternberg et al., 2014). In 2014, genome-wide chromatin-immunoprecipitation sequencing (ChIP-seq) was used to map the binding sites of catalytically inactive Cas9 (dCas9). The results validated the PAM requirements for Cas9 binding and showed that targeting was enriched in open chromatin loci, indicating the chromatin structure as one factor mediating Cas9 sequence recognition (Kuscu et al., 2014). The latter finding was independently validated afterward (Yarrington et al., 2018; Janssen et al., 2019).

## Off-Target Prediction Tools

**FIGURE 3:**
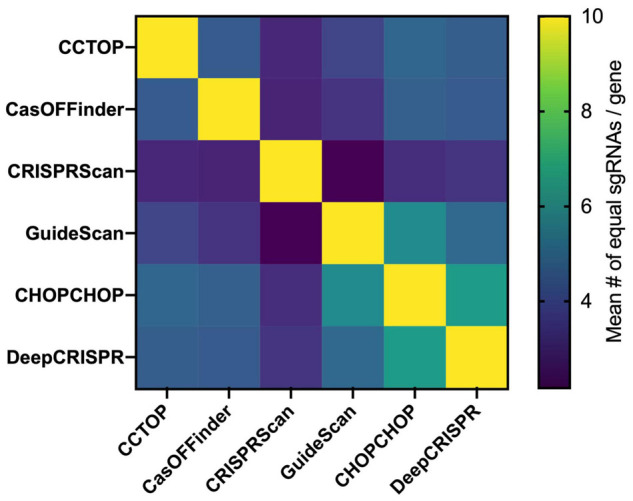
sgRNA choice comparison between available online tools. Using a total set of 36 exons from different genes with an average size of 386 ± 530 bp (list below), the top 10 sgRNA of the CCTop, CasOFFinder, CRISPRScan, GuideScan, CHOPCHOP, and DeepCRISPR were determined. Afterward, the common top sgRNAs between any two tools were identified for each, and the mean number of common guides is shown in the matrix based on a heatmap. Each square represents the comparison of the corresponding horizontal and vertical position. The different choice of sgRNAs between the available tools for a given genomic region target is associated to the choice of different collections of potential off-targets. The list of genes used: *SPHK1* (Hs), *SPHK2* (Hs), *IL7RA* (Hs), *IDE* (Hs), *HLA* (Hs), *BLM* (Hs), *GUSB* (Hs), *AZIN1* (Hs), *ADRA2C* (Hs), *ADRB2* (Hs), *MAPT* (Hs), *SMAD3* (Hs), *EIF2AK4* (Hs), *MVK* (Hs), *Anxa1* (Mm), *Adra2c* (Mm), *KLHL* (Hs), *Mlh1* (Mm), *Msh6* (Mm), *KRAS* (Hs), *DHX37* (Hs), *RYR2* (Hs), *NR3C1* (Hs), *ODF2* (Hs), *Hnf4a* (Mm), *Braf* (Mm), *POLMU* (Hs), *LYPLA2* (Hs), *PPT1* (Hs), *ESRRA* (Hs), *TCRB* (Hs), *BTBD9* (Hs), *GALNT1* (Hs), *MAN2A* (Hs), and *ST6GAL1* (Hs).

It is not possible to completely avoid off-targets, but selection of the on-target sgRNAs with the highest efficiency and the smallest number of off-targets or without off-targets in critical genomic regions, such as coding sequences, can reduce off-target frequency/effects. As of the writing of this review, 49 online tools can predict off-target sequences and score the gRNA efficiency for on-target activity (Torres-Perez et al., 2019). These tools can be alignment- or scoring-based. Alignment-based methods rely on the sequence similarity search of the sgRNA sequence in a given genome and, according to the mechanistic studies on Cas9-mediated DSB sequence recognition requirements, off-target sites are determined. Bowtie, a pre-CRISPR-era NGS-based alignment tool (Langmead et al., 2009), allows very few mismatches in the sgRNA seed sequence, making off-target prediction incomplete (Doench et al., 2016). However, using the Bowtie algorithm, with modifications, the CCTop tool finds off-targets with up to five mismatches in the protospacer sgRNA sequence, and scores each sgRNA according to the likelihood of a stable sgRNA/DNA heteroduplex (Stemmer et al., 2015). CasOFFinder includes no limits in the mismatch base number (Bae et al., 2014). Both tools allow the choice of PAM sequences for different programmable nuclease platforms, but compared to experimentally generated off-targets, the CCTop and CasOFFinder tools only identify a subset of sites, suggesting that sequence similarity alone is not sufficient for complete off-target prediction (Cameron et al., 2017). Scoring-based methods include sgRNA whole-genome alignment and an additional score for each possible sgRNA. According to empirical data, scores are usually attributed to genome context (open vs. closed chromatin, for instance) and specific mismatch type or location within the protospacer. The MIT score, proposed in the early days of the CRISPR era, was derived from the activity evaluation of > 700 sgRNAs in > 100 predicted off-target loci. The data were used to determine a weight matrix for each mismatch position (Hsu et al., 2013). This score was integrated into several tools, such as CHOPCHOP (Labun et al., 2016) and CRISPOR (Haeussler et al., 2016). Another widely used scoring method is the Cutting Frequency Determination (CFD), which was generated from the analysis of the on and off-target activity of a > 27 k sgRNA library. The library included a primary set of all sgRNAs against *Cd33*, regardless of PAM, combined with variant sgRNAs having 1-nucleotide insertions, deletions, and mismatches (Doench et al., 2016). The CFD score is included in the CRISPRScan (Moreno-Mateos et al., 2015), CRISPOR (Haeussler et al., 2016), and GuideScan (Perez et al., 2017) tools. Finally, using empirical datasets, machine-learning-based tools have also been developed. DeepCRISPR provides off-target prediction considering epigenetic sequence factors and was reported to outperform the other available off-target prediction tools (Chuai et al., 2018). Scanning a given single exon from each of 36 different genes, it can be seen that there are major differences in the sgRNA choices of six widely used online tools (Figure 3), which means that these programs also select different constellations of off-targets, particularly in knockout projects, which impose fewer constraints in the selection of the sgRNAs than knockin projects. Systematic comparisons between the tools and methods for sgRNA determination are still needed.

## Detection of Off-Targets: Biased and Unbiased Methods

Bacteria have evolved for millions of years with their CRISPR systems. In contrast, human cells were exposed to Cas9 during evolution, and their genomes are orders of magnitude larger than those of bacteria. Thus, CRISPR off-target events are unavoidable in large Eukaryotic genomes, and their detection poses several technical challenges. Two types of detection methods have been used. In the biased methods, only sequences predicted by the *in silico* tools mentioned above or resulting from empirical studies are analyzed. This produces a relatively small set of potential off-target sequences that several methods can then analyze, including the detection of mispairings in the amplified region by the T7 endonuclease I, Sanger sequencing, Next Generation Sequencing (NGS), or off-target reporters (Table 1).

**TABLE 1:** Summary table of the methods currently available to detect off-target activity in gene editing applications.

Methods			References
	
Biased	Advantages	Disadvantages	
T7 endonuclease I	Fast and easy to use	Very limited to predict off-targets	
Sanger sequencing	Relatively fast and easy to use	Limited to amplified regions and low resolution when used in bulk cultures	
Next generation sequencing	High coverage and resolution	Not targeted to off-target discovery (unless it’s a whole-genome approach)	
Off-target reporters	Precise measure of off-target activity	Restricted to the reporter sequence	
**Unbiased**			
***In vitro***			
Digenome-seq	Detection of off-targets with a 0.1% or lower frequency of indels	High background of random DSBs, requires reference genome, expensive	Kim et al., 2015
SITE-Seq	Allows the recovery of both high- and low-cleaveage off-target sites	High background of random DSBs	Cameron et al., 2017
CIRCLE-seq	Background of RNP-independent genomic breaks is vastly reduced	Limited to cells that can be transfected with the GUIDE-seq dsODN tag	Tsai et al., 2017
**Chip-seq**			
Chip-seq (with dCas9)	Excellent coverage	Detects only dCas9-associated off-targets	Kuscu et al., 2014
DISCOVER-Seq	Allows the identification of the nuclease cutting site with a single-base resolution	Detects only off-targets associated with adenoviral-delivered Cas9	Wienert et al., 2019
**DSB capture**			
GUIDE-Seq	Can identify off-targets that are not detected by existing computational methods or CHIP-seq	Efficiency is limited to chromosomal relaxation	Tsai et al., 2015
HTGTS	Able to detect a wide range of broken end structures with nucleotide-level resolution. Sensitive and reproducible, relatively inexpensive and scalable	Only detects DSBs that translocate	Hu et al., 2016
IDLV	Efficient detection of off-targets in primary cells	Lower sensitivity than other methods	Wang et al., 2015
BLISS	Allows the direct labeling of DSBs in fixed cells or tissue sections on a solid surface, highly sensitive, easy scalability, and multiplexing	Detection of off-targets present only in the labeling period	Yan et al., 2017
VIVO	Detects *in vivo* off-target effects of gene-editing nucleases. Can be used with Cas9 variants and other Cas orthologs	−	Akcakaya et al., 2018

Given the current limitations and disparities in the results produced by the *in silico* approaches to detect the efficiency of the sgRNAs for their on-targets, the prediction of the off-targets is even more problematic, due to the increased number of variables and the lack of data for most species on the epigenetic features that are known to influence the specificity and efficiency of CRISPR editing, explored by programs such as DeepCRISPR (Chuai et al., 2018). Thus, the strategies of the second type, known as the unbiased methods, have gained crucial importance. The unbiased methods teach us about the best criteria to identify off-targets and provide a comprehensive collection of the real off-targets. There is now a plethora of techniques that we divide here into *in vitro*, ChIP-seq, and DSB-capture methods (Table 1).

One of the *in vitro* methods is Digenome-seq, in which the targeted cells are subjected to whole-genome sequencing. The genomic DNA is digested *in vitro* with the ribonucleoprotein complexes (RNPs) to maximize the detection of off-targets, which are then detected by NGS as fragments with identical 5′ ends, allowing the detection of off-targets with a 0.1% or lower frequency of indels (Kim et al., 2015). The authors concluded that Cas9 could be highly specific, inducing indels at only a few off-targets. Another *in vitro* method providing the same type of data is SITE-Seq, which is based on the cleavage of genomic DNA by an RNP and then the tagging, enrichment, and sequencing of the fragments (Cameron et al., 2017). One of the limitations of Digenome-seq and SITE-Seq is the high background of random DSBs. Thus, Circularization for *In vitro* Reporting of CLeavage Effects by sequencing (CIRCLE-seq) was eventually developed to solve this problem. Essentially, in this *in vitro* method the DNA is first circularized and then linearized by the RNP being tested. Since only these linearized products will be sequenced, the background of RNP-independent genomic breaks previously present is vastly reduced (Tsai et al., 2017).

ChIP-seq using a catalytically inactive Cas9 revealed that the potential off-targets are enriched in the open chromatin regions, and the number of off-targets varied widely from around 10 to over 1,000 depending on the sgRNA used (Kuscu et al., 2014). DISCOVER-Seq is another ChIP-seq approach, but based on the tracking of the MRE11, a repair factor that is recruited to the Cas9 cut site and allows the identification of the nuclease cutting site with a single-base resolution (Wienert et al., 2019).

Unlike the ChIP-seq methods, the key step in GUIDE-seq is the ligation to the DSBs of double-stranded oligodeoxynucleotides in live cells expressing the RNP followed by NGS (Tsai et al., 2015). It was reported that the majority of off-targets detected had not been detected by the computational methods available at the time. Another method based on the capture of the RNP-dependent DSBs is High-Throughput Gene Translocation Sequencing (HTGTS), which was originally developed to detect DSBs introduced by natural processes in lymphocytes (Hu et al., 2016). Unlike the previous method, in HTGTS the DSBs to detect are captured *in vivo* by a single DSB generated in the genome by a meganuclease. One additional DSB-capture method is based on the observation that the linear double-stranded genome of an integrase-defective lentiviral vector (IDLV) incorporates preferentially into DSBs by NHEJ. A lentivirus encoding a puromycin-resistant gene was used to tag *in vivo* the DSBs introduced by distinct RNPs and, after selection of the cells with puromycin, the integration sites of the lentivirus were mapped (Wang et al., 2015). Another technique, Breaks Labeling *In Situ* and Sequencing (BLISS), directly tags the DSBs in fixed cells or tissue sections (Yan et al., 2017). It should be noticed that these capture methods do not distinguish between the RNP-dependent breaks and other DSBs, which explains why there was only a 2–3 fold increase in the number of puromycin-resistant colonies relative to the control in the IDLV study (Wang et al., 2015). It is only by mapping the breaks to the genome more likely to be associated with the RNP activity that the off-targets are identified, which makes these capture methods not absolutely unbiased.

Verification of *in vivo* off-targets (VIVO) is a final method worth mentioning because it combines the specificity of the *in vitro* CIRCLE-seq to identify potential off-target sites with the NGS analysis of these regions in cells exposed to RNPs (Akcakaya et al., 2018). Interestingly, the study found that depending on the choice of the sgRNA, the off-targets could be substantial in number or undetectable.

## Strategies to Decrease Off-Target Activity Based on sgRna Engineering

Computational prediction of off-target activity is the first step in minimizing these undesired events. Additional experimental approaches have been proposed to ablate Cas9 off-target activity, based on several aspects of the CRISPR/Cas9 genome editing biology. The first evidence on the sgRNA-target DNA base pairing requirements, mentioned above, inspired initial work on off-target minimization. It was reasoned that truncated sgRNAs would reduce the RNA-DNA interface, increasing the Cas9/sgRNA complex binding sensitivity to mismatches, thus reducing off-target events. The use of truncated sgRNAs reduced the DSB generation in off-target sites by 5,000-fold compared to full-length ones (Fu et al., 2014). Reducing the length of the sgRNA also increases the potential number of off-targets, but 97 potential off-targets were analyzed, including all with a single mismatch, and only one had a detectable number of off-targets. Furthermore, the addition of two guanine nucleotides to the 5′ end of the complementary sequence of the sgRNA improved target specificity, reducing off-target events (Cho et al., 2014). More recently, the addition of a hairpin to the 5′ end of the sgRNA was shown to elevate the energetic requirements for sgRNA/target DNA base pairing, improving the specificity of the Cas9/sgRNA complex, thus decreasing the off-target activity (Kocak et al., 2019). The chemical modification of the ribose-phosphate backbone of a specific site within the sgRNA was also described to reduce off-targets (Ryan et al., 2018).

## Engineered Cas9 Variants to Decrease Off-Target Activity

*Streptococcus pyogenes* Cas9 (SpCas9) is the most broadly used Cas9. Initial studies employed mutated SpCas9 proteins, that work as nickases (nCas9), cleaving only one strand of DNA. To increase the complexity of the sequence that defines the target region, two RNPs with proximal protospacers would theoretically minimize off-target activity. In fact, two sufficiently close DNA strand nicks were able to generate DSBs (Ran et al., 2013) and were shown to decrease off-target activity (Ran et al., 2013; Shen et al., 2014). The use of two nCas9s improved specificity, since the off-targets for each sgRNA are unlikely to be close to each other and isolated off-target nicks are easily repaired. Two back-to-back papers (Guilinger et al., 2014; Tsai et al., 2014) also explored the concept of DSB generation dependent on two sgRNAs using a dCas9 fused to a *Fok*I nuclease to induce DSBs. In both studies, there was a substantial decrease in off-target activity when the dCas9-*Fok*I fusions were used. The peptide linkers between dCas9 and *Fok*I nuclease were then improved, and by deep sequencing no detectable off-target activity was found. Following these studies, chimeras of RNA-guided *FokI* nucleases have been produced that maintain high editing efficiency, solving the main limitation of this system (Havlicek et al., 2017).

One of the key developments for improved Cas9 proteins was its crystal structure resolution when in complex with the sgRNA and target DNA (Nishimasu et al., 2014). With the protein conformation information, it was possible to determine potential sites for the splitting of Cas9, enabling the controlled reassembly and activation of the protein. The use of this strategy showed no detectable off-target modifications by sequencing of predicted sites but diminished on-target activity (Zetsche et al., 2015b). The crystal structure also enabled the detection of a positively charged groove responsible for stabilizing the non-targeted DNA strand in the Cas9:sgRNA/DNA complex. Upon charge neutralization of the protein domain, Cas9:sgRNA binding to double-stranded DNA was more dependent on Watson-Crick base pairing between the sgRNA and the target DNA sequence, thus reducing the off-target activity to levels undetectable by whole-genome deep sequencing. This structure-guided engineered Cas9 was coined “enhanced specificity” SpCas9 (eSpCas9) (Slaymaker et al., 2016). In the same year, several amino acids of SpCas9 were identified to have non-specific DNA contacts with the target DNA strand, and once mutated, a decreased tolerance for protospacer mismatches was observed. Moreover, using GUIDE-seq (Tsai et al., 2015), off-target activity was not detected for the mutated SpCas9, named high-fidelity SpCas9 (SpCas9-HF1) (Kleinstiver et al., 2016a). Although highly specific, these two engineered SpCas9 proteins showed reduced on-target activity in some target genes (Kim S. et al., 2017). The mechanism for the increased specificity of these highly specific SpCas9 variants was later found to be due to raised thresholds for conformational activation upon DNA binding of these proteins. With this knowledge, a hyper-accurate Cas9 (HypaCas9) variant was developed that shows improved on-target specificity without compromising on-target activity (Chen et al., 2017). The Sniper-Cas9 variant was then developed using a directed evolution approach, revealing on-target activity level similar to that of SpCas9 and better specificity than the other high-fidelity engineered variants (Lee et al., 2018). Finally, a HiFi Cas9 variant was generated using an unbiased bacterial screen. It contains a single point mutation in the SpCas9 backbone that increases the on-target activity compared to the other rationally engineered variants, maintaining no detectable off-target activity (Vakulskas et al., 2018).

The race for the generation of Cas9 variants with the desired high on-target and negligible off-target activity appears to still be on a fast pace (Table 2). Further detailed comparisons between these and future engineered Cas9 variants are needed across genomes, delivery methods, and cell types.

**TABLE 2:** Summary table on the Cas9 nucleases currently available for CRISPR/Cas9-mediated gene editing.

Cas9 nucleases			

Engineered Cas9 variants	Characteristics	Off-target activity	References
SpCas9	Most broadly used Cas9		Jinek et al., 2012
nCas9	Mutated SpCas9 D10A protein (deactivation of the RuvC domain). Only cleaves one strand of DNA	1,500 × reduction of off-target effects	Ran et al., 2013
dCas9-*Fok*I	Catalytically inactive Cas9 (D10A and H840A) fused with *Fok*I catalytic domain	No detectable off-targets	Guilinger et al., 2014; Tsai et al., 2014
eSpCas9	Structure-guided engineered Cas9 with the point-mutations K848A, K1003A, and R1060A	Reduced off-target activity to non-detectable levels	Slaymaker et al., 2016
SpCas9-HF1	Mutated version of SpCas9 (N497A, R661A, Q695A, and Q926A)	Off-target activity not detected using GUIDE-seq	Kleinstiver et al., 2016a
HypaCas9	Hyper-accurate Cas9 variant (N692A, M694A, Q695A, and H698A mutations in the REC3 domain)	Better genome-wide specificity relative to both SpCas9-HF1 and eSpCas9	Chen et al., 2017
Sniper-Cas9	Developed using a directed evolution approach	Better specificity than the other high-fidelity engineered variants	Lee et al., 2018
**Cas9 orthologs**			
SaCas9	Ortholog derived from *Staphylococcus aureus*	Recognizes a larger PAM sequence, which theoretically improves specificity	Kumar et al., 2018
St1Cas9	Ortholog derived from *Streptococcus thermophilus*	Evaluation by deep sequencing shows lower off-target activity	Müller et al., 2016
St3Cas9	Ortholog derived from *Streptococcus thermophiles*	Evaluation by deep sequencing shows lower off-target activity	Müller et al., 2016
NmCas9	Ortholog derived from *Neisseria meningitides*	Recognizes a larger PAM sequence and has lower off-target activity compared to SpCas9	Lee et al., 2016
CjCas9	Ortholog derived from *Campylobacter jejuni*	Lower off-target activity compared to SpCas9	Kim E. et al., 2017

## On- and Off-Target Activity of Cas9 Orthologs

Since the early days of CRISPR research in bacteria, it was clear that this molecular immune system is ubiquitous (Gasiunas et al., 2020). As Cas9 orthologs began to be discovered and characterized, new tools were developed to induce programmable DSBs, with key differences from the SpCas9-based ones. The SpCas9 ortholog derived from *S. aureus* (SaCas9) has the advantage of being encoded by a small gene (SpCas9 4.1 kb, SaCas9 3.2 kb), which allowed the use of an adeno-associated-virus (AAV) vector for more efficient *in vivo* or *ex vivo* applications (Kumar et al., 2018). Moreover, SaCas9 recognizes a considerably larger PAM sequence than SpCas9, NNGRRT, which theoretically improves specificity. In fact, its application in human cell lines revealed absent off-target activity, evaluated by deep sequencing (Wang et al., 2019). Cas9 proteins from *S. thermophilus*, St1Cas9, and St3Cas9, specific for the PAM sequences NNAGAAW and NGGNG, respectively, also show lower off-target activity evaluated by deep sequencing (Müller et al., 2016). The SpCas9 ortholog from *N. meningitidis* (NmCas9) recognizes a larger PAM sequence, NNNNGHTT, and has lower off-target activity than SpCas9 (Lee et al., 2016). The *C. jejuni* Cas9 (CjCas9) is encoded by an even smaller gene than SaCas9 (2.9 kb) has specificity for two PAM sequences, NNNNACAC and NNNNRY, and lower off-target activity compared to SpCas9 (Kim E. et al., 2017). Despite these lower off-target activities, one should keep in mind that the higher PAM complexity of the less used Cas9 orthologs reduces the number of potential on-targets for a specific application.

Although Cas9 has dominated the gene-editing field, Cas12a (initially described as CpfI), which was cloned from *Francisella novicida*, has also been used as a tool. Cas12a and Cas9 belong to the class 2 CRISPR-Cas system, but the former has distinctive features, namely a smaller size, the natural use of a single RNA in the RNP complex, a T-rich PAM, and the production of a staggered DSB instead of the blunt break produced by Cas9 (Zetsche et al., 2015a). These breaks were shown to be advantageous for HDR-mediated genome editing (Moreno-Mateos et al., 2017). However, compared to Cas9, the slightly more complex PAM and reports of a lower efficiency (Lee et al., 2019) make it a less appealing tool. More importantly for this review, two careful independent analyses have shown that Cas12a introduces fewer off-targets than Cas9 (Kim et al., 2016; Kleinstiver et al., 2016b; Yan et al., 2017). Biochemical analyses suggest that this difference probably occurs because Cas12a is less tolerant to mismatches along most of the DNA target sequence than Cas9 (Strohkendl et al., 2018). However, *in vitro*, Cas12a and Cas9 have been shown to have strong nicking of DNA sequences containing up to four mismatches, non-specific sequence nicking of dsDNA when Cas12a is bound to a target DNA (Fu et al., 2019; Murugan et al., 2020), and RNA-independent, non-sequence specific DNA cleavage activities (Sundaresan et al., 2017). Whether these effects also occur *in vivo* in Eukaryotic cells is unclear, and nicks are typically less problematic than DSBs, but the sequence non-specific editing would be difficult to avoid using bioinformatic tools. Nevertheless, the discovery of different CRISPR systems is still a highly attractive subject of research and has expanded the tool kit for genome editing.

## Chromosomal Contexts and Off-Target Activity

Chromatin accessibility conditions the efficiency of the CRISPR system in gene editing; notably, the sgRNA binding is facilitated in open chromatin regions (Kuscu et al., 2014; Wu et al., 2014), and the state of the chromatin is often cell cycle-dependent. In addition, HDR is active predominantly during the S and G2 phases of the cell cycle. Thus, it is not surprising that the cell cycle influences both the CRISPR efficiency and the frequency of off-targets. The CRISPR-based adaptative immune system in bacteria has never had to tackle the challenge of DNA accessibility, as bacteria do not organize their genome in nucleosomes and archaea, despite having histones, maintain a relaxed structure allowing CRISPR to recognize phage DNA even if integrated into its DNA. More importantly, the foreign invading viral or phage DNA is highly accessible before encapsulation, allowing an ample window of opportunity for CRISPR-mediated immunity.

The efficiency of Cas9 editing is reduced in heterochromatic regions (Jensen et al., 2017). Both binding and cleavage are inhibited by nucleosomes (Horlbeck et al., 2016), particularly if the PAM region is located within the nucleosome core (Hinz et al., 2015; Isaac et al., 2016). This has been shown to occur both in human and plant cells (Liu et al., 2019), but not in zebrafish (Moreno-Mateos et al., 2015).

Therefore, quiescent cells or cells otherwise in a state of compacted chromatin hamper the efficiency of targeting in a way that may not be predictable by programs, as nucleosomes sequester putative cleavage sites, particularly on mismatch hits (Yarrington et al., 2018). On the other hand, the off-target effects of Cas9 are greatly reduced in chromatin regions (Hinz et al., 2015; Horlbeck et al., 2016), suggesting that the concerns raised by results of cell-free methods used to compile a list of potential off-target sites are greatly exaggerated, as many of these sites are inaccessible *in vivo* (Kim and Kim, 2018).

Some effort is being put into altering the chromosome accessibility to improve Cas9 activity *in vivo*. One strategy, proxy-CRISPR, consists of using a catalytically dead SpCas9 to bind to locations close to the target region, thus altering the local chromatin structure and exposing previously inaccessible targets to the nuclease activity of FnCas9, CjCas9, NcCas9, and FnCpf1; this strategy was shown to improve editing efficiency on these sites greatly and to lower the off-target activity (Chen et al., 2017). However, this method requires the expression of two CRISPR/Cas systems, making its *in vivo* application difficult. Another method explores Cas9 orthologs fused to chromatin-modulating peptides (CMPs), using a direct approach to remodel the chromatin of the target sites. This method greatly improves editing in refractory sites (Ding et al., 2019). However, for this technique to be used in clinical trials, it has to be determined if the presence of CMPs alters gene expression profiles. A more recent approach uses histone deacetylase (HDAC) inhibitors to promote chromatin accessibility for genome editing. Among the HDAC inhibitors tested, vorinostat (suberoylanilide hydroxamic acid) were the most efficient, raising both NHEJ- and HDR-driven editing, with notable efficiency in the increase of HDR in closed loci (Zhang et al., 2021). The observed increase of NHEJ may hinder tailored gene editing applications, and should be further analyzed.

Besides chromatin accessibility, the repair systems used by Eukaryotic cells change during the cell cycle. HDR is mainly active in mammalian cells during the S phase, when DNA replication leads to the generation of endogenous repair templates, and declines in the late-G2 phase. In contrast, the NHEJ pathway occurs in all phases with special emphasis to G1 (Mao et al., 2008). Therefore, knocking in a DNA sequence tends to be more efficient during the S and G2 phases. Moreover, one can expect that off-target DSBs generated in the presence of a more proficient HDR pathway are repaired with an endogenous sequence, thus decreasing the frequency of undesired mutations.

A way to increase the use of HDR to deal with the Cas9-induced site-specific DSBs is to synchronize the cells to deliver the Cas9 RNPs at a specific cell cycle phase. Cas9 RNP-mediated editing starts 4 h after nucleofection and lasts for 24 h until the RNPs are degraded (Kim et al., 2014). A careful study determined at which point cells should be synchronized to maximize HDR efficiency while reducing off-target effects. The most effective cell synchronization treatment was Nocodazole, which blocks the cells at the M phase. At this phase, cells have their DNA fully replicated, and their nuclear membrane is broken down (Lin et al., 2014). Researchers have also fused Cas9 to the N-terminal region of Geminin protein, a substrate for the E3 ubiquitin ligase complex APC/Cdh1. This strategy is particularly useful because it does not require cell cycle inhibitors, as it uses the cell cycle regulation machinery, marking Cas9 for degradation via ubiquitination during the G1 phase, thus ensuring the proper expression in time for the maximum HDR potential (Gutschner et al., 2016). More recently, the discovery of bacteriophage proteins that evade prokaryotic adaptive immunity, known as anti-CRISPR proteins (reviewed in Maxwell, 2017), allowed for a similar approach by fusing the anti-CRISPR protein AcrIIA4 to the Chromatin licensing and DNA replication factor 1 (CDT1), which ensures that DNA replication occurs only once per cell division. Gene editing is deactivated by the anti-CRISPR during the G1 and S phases, and during the G2 phase, because the AcrIIA-CDT1 fusion is degraded, Cas9 becomes active. This provided not only a decrease of the off-target effects but, at the same time, a fourfold increase in HDR efficiency (Matsumoto et al., 2020). The efficiency was higher than in previous studies as the Cas9 is only inactivated but not degraded during the G1 phase.

A different strategy is to shift the balance of NHEJ vs. HDR by disrupting key NHEJ players, either using molecular inhibitors, short hairpin RNAs (shRNA), or the co-expression of Cas9 with proteins that mediate the proteasomal degradation of ligase IV (Chu et al., 2015). SCR7 is a ligase 4 inhibitor that has been used in numerous studies with inconsistent results, suggesting that its action is cell-type- or gene-dependent, making it a poor general promotor of HDR for CRISPR (Bischoff et al., 2020). A small molecule, RS-1, which stimulates RAD51 and therefore HDR, improved the efficiency of targeted integrations (Song et al., 2016); other small molecules have been shown to promote HDR directly or indirectly by reducing NHEJ (reviewed in Bischoff et al., 2020). The use of small molecules has also been suggested to help attenuate the off-target problem. For example, by inhibiting NHEJ, the frequency of off-targets is greatly reduced (Zheng et al., 2020), whereas by boosting the HDR efficiency with small molecules targeting can be successfully achieved in diverse cell types with limited toxicity. These techniques open new venues for therapeutic uses in cells that may be more prone to off-target effects than iPSCs. The inhibition of NHEJ could be, however, a source of greater genomic instability in CRISPR applications.

## Off-Target Activity According to Cell Type

The idea that the cell type might influence off-target activity is based on both the evidence that the on-target effects vary with cell type mostly due to chromatin condensation profiles and DSB repair has different efficiencies depending on the cell type. Using ChIP-seq to perform an unbiased genome-wide detection of Cas9 binding sites for different sgRNAs, it was found that open chromatin regions are enriched for off-target sites (Kuscu et al., 2014; Wu et al., 2014). In addition, a pioneer study has sparked the discussion by finding different potential off-target binding in HeKaS3 vs. HEK293T cells. Off-target events might depend on several factors beyond the DNA sequences, including the cell type, DNA-RNA heteroduplex, R-loop expansion, and so on (Duan et al., 2014). More importantly, for translational applications of the technique, the off-target potential of Cas9 has proven to be very limited in human iPSCs, where the DSB repair pathways are working normally, when compared to regular cell lines (Schwank et al., 2013; Smith et al., 2014; Veres et al., 2014).

Another cell-dependent mechanism studied for its effect on Cas9 is CpG site methylation, a cell-type-specific epigenetic mechanism. Despite an early study stating that CpG methylation and chromatin accessibility have little effect on targeting (Hsu et al., 2013), a subsequent analysis determined that CpG sites are negatively correlated with Cas9 binding, particularly when mismatches are present (Wu et al., 2014). We now know that the off-target potential is cell-type based due to the DSB repair system, the state of chromatin condensation, and the methylation pattern of CpG sites.

## Off-Target Activity According to Delivery Type

Two back-to-back publications showed that the delivery of RNPs to target cells have reduced off-target effects compared to the use of plasmids encoding Cas9 and sgRNA (Kim et al., 2014; Ramakrishna et al., 2014). RNP delivery was performed using cationic lipid-mediated delivery (lipofectamine) and positively charged nanoparticles, leading to the same results. The RNP binds and cleaves the chromosomal DNA after delivery and is quickly degraded (Banas et al., 2020), leaving little time for the less efficient off-target cleavage, whereas the plasmid expression subsists for days before degradation. The ratio of off-target to on-target mutations has been calculated to be 28-fold lower in RNPs compared to the plasmid-based methods (Lu et al., 2019). In addition, the RNP dose has been calculated to be lower in the RNP complex transfection, resulting in lower cytotoxicity. Lipofectamine delivery was confirmed to work *in vivo* in the inner ear of mice (Zuris et al., 2015), and positively charged nanoparticles (lipidoids) were also confirmed to deliver the editing tools to the brain of mice (Wang et al., 2016).

Plasmid expression requires nuclear transport for RNA transcription and mRNA export for translation. The expressed Cas9 must then complex with the sgRNA and be transported into the nucleus for the editing to occur. To complete these steps, it has been calculated that approximately 24 h are required (Shen et al., 2019). This time can hinder some experiments dependent on synchronized drug delivery or template donor delivery for HDR. The delivery of Cas9 may also be in the form of RNA for direct translation or protein (ready to work in a ribonucleoprotein complex composed of Cas9 and sgRNA); RNA overcomes the risk of integration and has poor stability compared to plasmid DNA.

Besides the delivery of Cas9 as a protein or as a plasmid, there are several delivery systems available. These can be grouped as viral vectors, physical (i.e., microinjection or electroporation), or chemical methods, (cationic-lipid transfection and lipid nanoparticles), ligand fusion tags, cell-penetrating peptides, and gold nanoparticles, among others. The most common viral vectors are adenoviruses, adeno-associated viruses, and lentiviruses, since these are the ones approved for clinical trials (Chakraborty, 2019).

Viral vectors may lead to unintended Cas9 genome integration, raising important questions on the safety of clinical trials. This topic remains a matter of debate (Chakraborty, 2019). There is also the risk of immunogenicity both from the use of the virus and the expression of the Cas9 protein. Even though the safety of viral approaches has been addressed and improved, the off-targets remain a concern due to the long-term exposure caused by the delivery method; furthermore, the viral vectors have limited packaging capacity.

An approach to avoid the risk of integration, immune response, and the long-term Cas9 expression has been created using a lentivirus-like particle (LVLP) encapsulating up to 100 copies of Cas9 mRNA per LVLP (Lu et al., 2019). Along the same lines, to limit the expression of the nuclease and reach a broad range of target cells, the Cas9/sgRNA RNP was recently packaged into extracellular nanovesicles, leading to an enormous reduction of off-targets compared to the plasmid-based delivery without loss of on-target cleavage (Gee et al., 2020).

## Off-Targets in Other Gene-Editing Technologies: Zfns, Talens, and Base Editors

Precise gene editing of mammalian cells precedes CRISPR-mediated editing by over 25 years, with the work of Capecchi (Folger et al., 1984) and Smithies (Smithies et al., 1985), who proved that homologous recombination could integrate genetic material. Despite this breakthrough, the low efficiency of integration requires a selection marker or the introduction of a DSB to stimulate DNA repair. Meganucleases of microbial origin capable of recognizing sequences of 14–40 bp can generate DSBs, but the collection of sequences recognized by these enzymes is limited. The ability to introduce a DSB in most regions of the genome emerged with the invention of the chimeric nucleases that use the cleavage domain of the type IIS restriction enzyme *Fok*I fused to modules of proteins that bind to specific DNA sequence. The first of these tools to appear were the Zinc Finger Nucleases (ZFN) (Geurts et al., 2009), in which the DNA specificity is given by the association of several finger domains, each contacting primarily with three specific bp of DNA. In the second type of chimeric nucleases, known as Transcription Activator-Like Effector Nucleases (TALENs) (Miller et al., 2011), the DNA binding specificity is given by the modules of transcription activator-like effectors of bacterial origin, each recognizing a specific bp. ZFNs and TALENs were shown to work with similar efficiencies in iPSCs (Hockemeyer et al., 2009, 2011).

A simple *Pubmed* search shows that the CRISPR technology is now vastly more popular within the research community than the ZFN or TALEN ever were, but in a clinical setting, ultimately, it is the safety of the method that will prevail. There is a recent review comparing these two techniques with CRISPR-mediated editing (Khalil, 2020). Thus, here we will compare the three techniques exclusively concerning the off-targets. In principle, both ZFN and TALEN should have a lower frequency of off-targets than the original CRISPR/Cas9 technique because the *Fok*I cleavage domain works as a dimer, which requires the targeting of two neighboring regions in the genome to produce a DSB, thus vastly decreasing the number of off-targets, as explained in a previous section. However, the comparison is not straightforward, due to the different complexity of the sequences targeted by each strategy and the different biochemistry of RNA:DNA and protein:DNA interactions. Nevertheless, both for the chimeric nucleases and CRISPR, increasing the interaction length with the target sequence did not improve the specificity or efficiency (Ran et al., 2013; Yan et al., 2013).

ZFN off-targets with up to three mutations relative to the canonical target sequence were identified using *in vitro* cell-free strategies and confirmed in cell lines and zebrafish (Gupta et al., 2011; Pattanayak et al., 2011). A thorough study using the unbiased IDLV-based methods to tag ZFN-dependent DSBs revealed several off-targets, but overall that ZFNs are highly specific, particularly when obligate heterodimeric *Fok*I domains are used. In a TALEN-engineered mouse, no off-targets were detected, although only low-resolution and limited direct methods were used (Gabriel et al., 2011). And a hybrid strategy combining a ZFN and a TALEN seems to improve specificity even further (Yan et al., 2013). Unfortunately, properly controlled studies directly addressing the off-targets produced by CRISPR and the chimeric nucleases are scarce. In a goat genome editing project, CRISPR/Cas9 was reported to have introduced more off-targets than a TALEN approach for the same editing (Zhang et al., 2019); in another set of experiments using IDLV tagging of DSBs, the frequency of off-targets generated by Cas9 and TALENs were directly compared and the results revealed that three different TALENs generated no off-targets, whereas three of six Cas9 RNPs had detectable off-target activity (Wang et al., 2015). Interestingly, besides detecting the known tolerance for a few mismatches, the authors found patterns suggesting that a single-base bulge formed between the sgRNA and the genomic target could also be tolerated. Thus, paradoxically, the data suggest that the wild-type SpCas9 that has been the focus of so much attention introduces more off-targets than the TALENs and, as mentioned above, Cas12a.

The dCas9 (catalytically null version of Cas9) has been fused to many proteins to become a molecular swiss-army knife capable of driving different enzymatic activities or useful structural properties (e.g., fluorescent proteins) to precise locations in the genome. Some of these strategies have also transformed the original Cas nuclease into base editors by fusing a dCas9 or a Cas nickase with natural cytosine base editors (capable of C-to-T conversions) or engineered adenine base editors (that catalyze A-to-G conversions) to generate single-base editors capable of editing close to a third of the annotated human pathogenic alleles (reviewed in Anzalone et al., 2020). The absence of a potentially dangerous DSB during the editing process is an attractive feature of these tools. However, they use deaminases that have been associated with mutational signatures in several types of cancers. A study in mouse embryos revealed that CRISPR-Cas9 and the adenine base editor had a frequency of mutations close to the spontaneous mutation rate; in contrast, cytosine base editing induced single nucleotide variants at more than 20-fold higher frequencies (Zuo et al., 2019). Thus, base editors have been fine-tuned in recent years through the titration of the expression levels, the use of a “high-fidelity” Cas9 partner (e.g., Rees et al., 2017; Hu et al., 2018; Lee et al., 2018; Doman et al., 2020) or the use of deaminases that preferentially deaminate cytidines in specific motifs (e.g., Gehrke et al., 2018). Nevertheless, besides the Cas9-dependent off-targets, these tools also produce Cas9-independent deamination events that are difficult to measure because of their random nature and rarity. This problem has been addressed independently by two groups, and these efforts include a thorough study of 153 deaminase domains to isolate and further engineer those with the lowest Cas9-independent off-target DNA deamination (Doman et al., 2020; Yu et al., 2020).

## Conclusion and Future Perspectives

Off-targets are not a problem for basic research using cell lines and animal models. It is sufficient to adopt the experimental design developed several years ago for shRNA studies to ensure that the observed effect is due to the on-target edition. In other words, if two clones obtained with different sgRNAs have the same phenotype, it is very unlikely that the effect is due to a technical artifact because the two guides will have different constellations of off-targets. In addition, rescue experiments are also possible and could be reassuring. It is only in the context of the ongoing clinical trials in humans using somatic cells and in the possible future edition of the germline that the off-targets remain a problem, although not the only one. That is the motivation fueling the many approaches to attenuate the off-target problem.

Two tragic stories haunt the field of gene therapy. Mr. Jesse Gelsinger died in 1999 from an exacerbated innate immune response to an adenoviral vector carrying a gene to correct his partial deficiency of ornithine transcarbamylase (Wilson, 2009). Not long after, out of 20 children suffering from X-Linked Severe Combined Immunodeficiency who were treated by gene therapy in two independent trials, five developed T cell leukemia, which would prove fatal in one case (Cavazzana et al., 2016); all cases resulted from the insertion of the retroviral vector carrying the correct form of the γc-encoding gene near tumor-promoting genes. The field eventually recovered from these early failures by developing safer vectors and protocols, and also novel technologies, such as CRISPR, that restore the correct DNA sequence and proper regulation. CRISPR gene editing is now regarded as a much safer gene therapy than the crude pioneering viral-based therapies, which were not gene editing approaches. However, any tragic clinical outcome associated with these novel technologies and whether the prior risk-benefit analyses need to be recalibrated.

CRIPSR-mediated gene editing has been described in models of Duchene muscular dystrophy (DMD), a genetic disease caused by mutations in the dystrophin gene (*DMD*), which encodes a cytoskeletal protein essential for muscle cell membrane integrity (Zhang et al., 2017). Cas12a-mediated gene editing was first employed in DMD patient-derived iPSCs and also in a murine model of the disease, with a mutation in Exon 51 of the *DMD* gene that generates a premature stop codon. The Cas12a-mediated gene editing rescued the disease phenotype in both models. The off-target activity was assessed by the T7 Endonuclease assay in 10 potential off-target sites, predicted by Cas-OFFinder, with no detected activity (Zhang et al., 2017). In a follow-up study, the same group applied the same strategy in a canine model of DMD in which the delivery of Cas9:sgRNA was done with adeno-associated viruses, via intramuscular injection (Amoasii et al., 2018). The disease phenotype was rescued with the application of CRISPR-mediated gene editing. Three off-target sites in coding regions were predicted using the MIT CRISPR design tool and deep sequencing of those sequences was performed. There were no genetic alterations in the predicted off-target sites (Amoasii et al., 2018). CRISPR/Cas9-based gene editing has been used in several other animal models of disease, particularly the murine ones. The off-targets have been measured using biased methods and Sanger sequencing of a small number of cloned amplicons from a few potential off-targets, such as in a model of Huntington’s disease (Monteys et al., 2017), biased methods and deep sequencing of amplicons from a modest number of potential off-targets, such as in a murine genetic model of amyotrophic lateral sclerosis (Gaj et al., 2017), and biased and unbiased methods (whole-exome and whole-genome sequencing), such as in a study of an Alzheimer’s disease model (Park et al., 2019). The overall picture is that off-targets and chromosomal rearrangements have not been detected at alarming frequencies in any CRISPR/Cas9-based gene editing to correct genetic deficiencies in animal models of disease. However, the methodology of off-target detection and quantification has not been standardized across these studies.

It is worth keeping in mind that clinical applications of the CRISPR technology in humans started a few years ago, even though the technique is still being perfected. In February 2019, the first report of cancer patients treated with CAR-T cells that had been edited using a combination of a retroviral vector and three CRISPR-knocked-out genes was published (Stadtmauer et al., 2020); although the immunotherapy itself did not provide complete cancer treatment, the engineered cells were well-tolerated. Another CRISPR technology application in the clinic was a phase I clinical trial of edited T cells, where the goal was to disrupt the *PD-1* gene to overcome the high expression of PD-L1 in the malignant cells of refractory non-small-cell lung cancer (Lu et al., 2020). In that study, the off-targets were determined using Cas-OFFinder and indels in those regions were detected by NGS. The off-target mutational frequency was 0.05% in 18 determined off-target sites, which was considered to be safe. Moreover, a CRISPR/Cas9 application in the treatment of the Transfusion-dependent ß-thalassemia and sickle cell disease (both severe monogenic diseases) was reported in January of 2021 (Frangoul et al., 2021). CD34+ hematopoietic stem cells were transfected with a Cas9:sgRNA complex targeting the *BCL11A* erythroid-specific enhancer. A total of 223 off-target sites were scanned, and no evidence of off-target activity was detected. Although these reports suggest that the off-targets may not be a problem for clinical applications, three potential technical limitations should be kept in mind. First, any long-term side-effects of the CRISPR editing, such as a slow-growing cancer, will remain undetectable for some time. Second, even when amplicon-based and hybridization capture steps are used to enrich the target genomic regions to be analyzed by NGS, this technique has a resolution limit, particularly when bulk cell populations are analyzed. Third, whole-genome sequencing of large collections of small pools of cells to evaluate in an unbiased way the frequency of off-targets is currently not possible to implement on a routine basis in clinical settings. Notwithstanding these limitations, there are over 60 ongoing clinical trials using CRISPR technology to treat more than 30 different diseases (see CRISPR News Medicine^1^). Standardized measures of off-target analysis in these trials would be welcome.

The many ingenious strategies developed over the last 8 years to decrease the frequency of off-targets that we have described have not exhausted the field. Recently, CRISPR GUARD was reported, a technique based on the targeting of the off-targets by RNPs with short RNAs that do not allow nuclease activity but can outcompete the on-target guide RNA at selected off-targets (Coelho et al., 2020). Although the practicability of this technique remains to be tested, it illustrates how truly novel ideas keep emerging. Such ingenuity will be required to tackle several remaining challenges, including: (1) the generation of improved *in silico* tools that integrate what is known about the tolerance for mismatches but also on single-base bulges and all the epigenetic features that influence the chances of a hit in an off-target; (2) more detailed and cell-specific analyses using unbiased methods of the off-targets in cells with therapeutic potential, such as human CD8 T cells and iPSCs of different sources; (3) a better integration of the strategies to reduce off-targets generated at several fronts and the implementation of standards to facilitate comparisons across different studies. This third point seems crucial. Although platforms such as Addgene have facilitated the sharing of molecular tools, it seems that the progress in the field has been delayed by the failure to implement two strategies. First, using the same cell line or animal models, engineered with reporters or not, and common protocol and readouts to compare the results of the different tools would provide quantitative comparisons that would quickly identify the best tools across different platforms. Second, despite the quest for innovation and commercial interests do not create incentives to fully explore the combination of methodologies already described that could lead to cumulative or synergistic decreases in the frequency of off-targets.

## Author Contributions

MMV, MC-F, and VMB wrote the manuscript, which was edited by JMPJ and JTP. JMPJ worked in the preparation of the data for Figure 2. MMV generated all the figures. All authors contributed to the article and approved the submitted version.

## Conflict of Interest

VMB runs a CRISPR-based gene editing service at Nova Medical School, a public university. The remaining authors declare that the research was conducted in the absence of any commercial or financial relationships that could be construed as a potential conflict of interest.

## Publisher’s Note

All claims expressed in this article are solely those of the authors and do not necessarily represent those of their affiliated organizations, or those of the publisher, the editors and the reviewers. Any product that may be evaluated in this article, or claim that may be made by its manufacturer, is not guaranteed or endorsed by the publisher.

## Footnotes

1
https://crisprmedicinenews.com/clinical-trials/
